# Remimazolam Anesthesia for Thyroid Surgery

**DOI:** 10.1155/2023/2352693

**Published:** 2023-05-15

**Authors:** Sae Nakagawa, Tomoharu Shakuo, Sakurako Matsudo, Hiroaki Soda, Kenji Shida

**Affiliations:** Department of Anesthesiology, Showa University Northern Yokohama Hospital, 35-1, Chigasaki-Chuo, Tsuzuki-Ku, Yokohama-Shi, Kanagawa-Ken 224-8503, Japan

## Abstract

**Background:**

Critical upper airway obstruction, hematoma formation, and recurrent laryngeal nerve palsy have been reported as postoperative complications of thyroid surgery. Although remimazolam may reduce the risk of these complications, the efficacy of flumazenil with remimazolam has not been reported. We present the successful anesthesia management of thyroid surgery using remimazolam and flumazenil. *Case Presentation*. A 72-year-old woman was diagnosed with a goiter and scheduled for a partial thyroidectomy under general anesthesia. We used remimazolam for induction and maintenance using a neural integrity monitor, electromyogram, and endotracheal tube under the bispectral index monitor. At the end of the surgery, spontaneous respiration was confirmed after the intravenous administration of sugammadex, and the patient was extubated under mild sedation. In the operating room, we administered flumazenil intravenously to confirm recurrent laryngeal nerve palsy and active postoperative hemorrhage. The patient was confirmed to have no recurrent laryngeal nerve palsy under full wakefulness but developed active postoperative hemorrhage with normal blood pressure. The patient required reoperation and was reintubated under intravenous administration of propofol. The anesthesia was maintained using 5% of desflurane, and the patient was extubated without any postoperative problems. The anesthesia was then terminated. The patient had no recall of the procedure.

**Conclusion:**

Maintenance of general anesthesia using remimazolam allowed the use of a neurostimulator with minimal muscle-relaxant effects, and extubation under sedation reduced the risk of abrupt and unexpected changes in blood pressure, body movement, and coughing. Furthermore, after extubation, the patient was rendered fully awake using flumazenil to confirm the presence of recurrent laryngeal nerve palsy and active postoperative hemorrhage. In addition, the patient had no memory of the reoperation, suggesting that the anterograde amnesic effect of remimazolam had a favorable psychological outcome associated with the reoperation. We safely managed thyroid surgery using remimazolam and flumazenil.

## 1. Introduction

Critical upper airway obstruction, postoperative hemorrhage/hematoma, and recurrent laryngeal nerve palsy are important complications of thyroid surgery [1] that may result in life-threatening complications. Therefore, anesthesia management should include the use of a neural integrity monitor (NIM) electromyogram endotracheal tube, an intraoperative neuromonitoring (IONM) technique that requires limiting muscle relaxants, minimize stimulation that could lead to postoperative hemorrhage during arousal, and ensure full arousal to confirm the absence of recurrent laryngeal nerve palsy and inadequate hemostasis [2–4].

Remimazolam is an ultra-short-acting benzodiazepine general anesthetic approved in Japan in 2020 for the induction and maintenance of general anesthesia [5]. We perform general anesthesia with remimazolam for partial thyroidectomy to reduce the risk of postoperative complications. We report this case because prompt detection of postoperative hemorrhage due to inadequate hemostasis could have prevented the development of a life-threatening hematoma in the patient while in the ward [1, 2, 4].

## 2. Case Presentation

The patient was a 72-year-old woman (height, 149 cm; weight, 67.0 kg) with no previous medical history who was diagnosed with an enlarged thyroid gland during a physical examination. Ultrasonography revealed a 42 × 27 × 59-mm goiter in the right lobe of the thyroid gland, and a partial thyroidectomy was scheduled. General anesthesia using remimazolam was planned to allow extubation under mild sedation and complete arousal after flumazenil administration.

**Figure 1:**
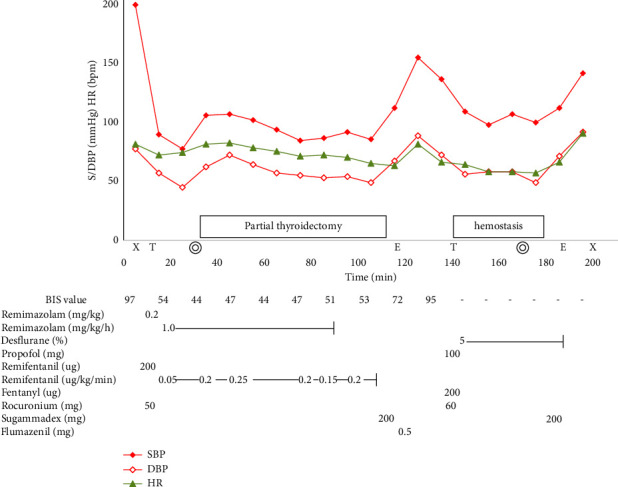
The anesthetic record. SBP: systolic blood pressure, DBP: diastolic blood pressure, HR: heart rate. X: start and end of anesthesia, T: intubation, E: extubation, double circle: start and end of surgery.

Upon arrival at the operating room, remimazolam (0.2 mg/kg), rocuronium (50 mg), and remifentanil (200 *μ*g) were administered intravenously for rapid induction, and intubation was performed using an NIM. Anesthesia was maintained using intravenous remimazolam (1.0 mg/kg/h) and remifentanil 0.2 (*μ*g/kg/min). The intraoperative bispectral index (BIS) ranged from 40 to 60. Administration of an antagonist of the muscle relaxant sugammadex to the NIM is often required after induction. However, in the present case, since nerve stimulation showed a normal response, the surgery was completed without using an antagonist. Rocuronium was not administered intraoperatively. The total operation time was 40 min, and the blood loss volume was 85 mL. Postoperatively, the patient was extubated after intravenous administration of 200 mg of sugammadex and oral and endotracheal suctioning, and the presence of spontaneous respiration and compliance was confirmed. Subsequently, flumazenil (0.5 mg) was administered intravenously, and full arousal was confirmed. The absence of recurrent laryngeal nerve palsy was confirmed by vocalization. No dyspnea was observed during inspiration. However, neck swelling was observed, and postoperative hemorrhage was suspected. Therefore, the decision was made to reoperate. Since the patient had already received flumazenil, propofol was selected as the sedative for reinduction. Anesthesia was quickly induced using 100 mg of propofol, 200 *µ*g of fentanyl, and 60 mg of rocuronium intravenously, and the patient was intubated using a normal tracheal tube. Although the patient had already received sugammadex, she was intubated without any complications with intravenous 60 mg of rocuronium. Anesthesia was maintained using 5% of desflurane. After the operation, the patient was extubated again after administering intravenously 200 mg of sugammadex, and anesthesia was terminated. Although complete arousal was not achieved, spontaneous respiration and handshake compliance were confirmed, and the patient was discharged (Figure 1).

During an interview on the day after the procedure, the patient had no recollection of the second intubation after the first extubation.

## 3. Discussion

After thyroid surgery, extubation and arousal of patients with minimal rapid blood pressure elevation and body movement are essential to prevent increased venous pressure, occurring due to coughing or retching subsequent to postoperative hematoma [4]. However, to check for recurrent laryngeal nerve palsy and active postoperative hemorrhage, vocalization and neck swelling should be confirmed under full arousal and normal blood pressure conditions [1].

In the present case, extubation under remimazolam sedation minimized the risk of postoperative hemorrhage caused by elevated blood pressure and coughing. Subsequent arousal using flumazenil allowed us to confirm the absence of recurrent laryngeal nerve palsy; however, active postoperative hemorrhage occurred due to inadequate hemostasis [4–7]. Remimazolam is rapidly hydrolyzed in the liver, mainly by carboxylesterase, and has an elimination half-time of 48–49 minutes [6]. Although narcotic overdose, residual muscle relaxants, and prolonged sedation can hinder the resumption of spontaneous breathing, in the present case, the rapid metabolism of remimazolam was ensured by antagonizing it with sugammadex 24 min after termination of administration, and spontaneous breathing was observed immediately. After extubation, spontaneous breathing was maintained, and the patient was sedated until a Richmond Agitation-Sedation Scale score of −2 was obtained. The surgeon requested full arousal of the patient to confirm vocalization; however, active postoperative hemorrhage was missed. Flumazenil was administered, and full arousal was achieved. Since flumazenil has a longer elimination half-life than remimazolam (60–77 minutes) [8], the risk of resedation after flumazenil administration is relatively low, and depending on the situation, the anesthesiologist can safely control the level of arousal using flumazenil after extubation, with no upper airway obstruction and spontaneous breathing. On the other hand, some reports have described patients who were fully awake after general anesthesia with remimazolam and received flumazenil but were resedated after returning to the room [9] and extubated after spontaneous breathing was confirmed without achieving subsequent arousal, resulting in multiple administrations of flumazenil [10]. When administering flumazenil, it is necessary to confirm that there is no loss of spontaneous respiration or upper airway obstruction after extubation, and careful observation is essential, considering the possibility of resedation. Other precautions to be maintained during the administration of flumazenil include monitoring for seizures and benzodiazepine withdrawal symptoms. Withdrawal symptoms such as agitation and hyperventilation may occur when flumazenil is administered to patients who have received long-term benzodiazepines, and convulsions may be induced in patients receiving anticonvulsants after the administration of benzodiazepines and subsequent antagonists with flumazenil [11]. In addition, flumazenil administration may cause antidepressant intoxication in patients taking tricyclic or tetracyclic antidepressants and benzodiazepines simultaneously [11]. Therefore, flumazenil should be used cautiously, with due consideration of the patient's medical history and medication intake.

In the present case, a nerve stimulator with an NIM was used for monitoring to prevent recurrent laryngeal nerve palsy, a complication of thyroid surgery [1, 12]. In such cases, if the intraoperative muscle-relaxant effect of the drug is affected during surgery, adequate nerve stimulation will not be achieved, and accurate monitoring may not be possible [1]. Therefore, a muscle-relaxant antagonist may have to be administered if the effect of rocuronium used during induction persists. In the present case, the patient showed a normal response to the neurostimulator, and sufficient stimulation was achieved without the need to administer a muscle-relaxant antagonist. Remimazolam may have fewer muscle-relaxant effects than inhaled anesthetics and may have a minimal effect on the neurostimulator [3, 13–19]. For the maintenance of anesthesia for thyroid surgery requiring neurostimulation, continuous administration of propofol, which has minimal muscle-relaxant effects, may be selected, but propofol infusion syndrome should be looked out for.

Reintubation after extubation, as in this case, is a psychologically taxing event for the patient; however, the present patient had no recollection of the second intubation after the first extubation. Anterograde amnesia with midazolam has already been reported, suggesting that remimazolam, which has a similar structure to midazolam, also has anterograde amnesic effects, which could have reduced the psychological burden on the patient and facilitated the reoperation [20, 21].

In the present case, remimazolam was used during thyroid surgery to modulate the effects of the neurostimulator and to manage anesthesia with attention to postoperative complications. Especially, early detection of postoperative hemorrhage occurring due to inadequate hemostasis is important, as it can be life-threatening if the patient develops a hematoma after returning to the ward. The amnesic effect of remimazolam also proved to be useful in addressing the psychological aspects associated with the reoperation. We present the successful anesthesia management of thyroid surgery using remimazolam and flumazenil.

## Data Availability

No data were used to support this study.

## Abbreviations

NIM: Neural integrity monitor
IONM: Intraoperative neuromonitoring.

## References

[1] Suzuki S., Yasunaga H., Matsui H., Fushimi K., Saito Y., Yamasoba T. (2016). Factors associated with neck hematoma after thyroidectomy: a retrospective analysis using a Japanese inpatient database. *Medicine (Baltimore)*.

[2] Randolph G. W., Dralle H., Abdullah H. (2011). Electrophysiologic recurrent laryngeal nerve monitoring during thyroid and parathyroid surgery: international standards guideline statement. *The Laryngoscope*.

[3] Bacuzzi A., Dionigi G., Del Bosco A. (2008). Anaesthesia for thyroid surgery: perioperative management. *International Journal of Surgery*.

[4] Edafe O., Cochrane E., Balasubramanian S. P. (2020). Reoperation for bleeding after thyroid and parathyroid surgery: incidence, risk Factors, prevention, and management. *World Journal of Surgery*.

[5] Masui K. (2020). Remimazolam besilate, a benzodiazepine, has been approved for general anesthesia. *Journal of Anesthesia*.

[6] Kilpatrick G. J., McIntyre M. S., Cox R. F. (2007). CNS 7056: a novel ultra-short-acting Benzodiazepine. *Anesthesiology*.

[7] Goudra B. G., Singh P. M. (2014). Remimazolam: the future of its sedative potential. *Saudi Journal of Anaesthesia*.

[8] Jones R. D., Chan K., Roulson C. J., Brown A. G., Smith I. D., Mya G. H. (1993). Pharmacokinetics of flumazenil and midazolam. *British Journal of Anaesthesia*.

[9] Yamamoto T., Kurabe M., Kamiya Y. (2021). Re-sleeping after reversal of remimazolam by flumazenil. *Journal of Anesthesia*.

[10] Takemori T., Oyama Y., Makino T., Hidaka S., Kitano T. (2022). Long-term delayed emergence after remimazolam-based general anesthesia: a case report. *JA Clin Rep*.

[11] Spivey W. H. (1992). Flumazenil and seizures: analysis of 43 cases. *Clinical Therapeutics*.

[12] Namizato D., Iwasaki M., Ishikawa M. (2019). Anesthetic considerations of intraoperative neuromonitoring in thyroidectomy. *Journal of Nippon Medical School*.

[13] Suzuki T., Munakata K., Watanabe N., Katsumata N., Saeki S., Ogawa S. (1999). Augmentation of vecuronium-induced neuromuscular block during sevoflurane anaesthesia: comparison with balanced anaesthesia using propofol or midazolam. *British Journal of Anaesthesia*.

[14] Bock M., Klippel K., Nitsche B., Bach A., Martin E., Motsch J. (2000). Rocuronium potency and recovery characteristics during steady-state desflurane, sevoflurane, isoflurane or propofol anaesthesia. *British Journal of Anaesthesia*.

[15] Paul M., Fokt R. M., Kindler C. H., Dipp N. C., Yost C. S. (2002). Characterization of the interactions between volatile anesthetics and neuromuscular blockers at the muscle nicotinic acetylcholine receptor. *Anesthesia & Analgesia*.

[16] Ye L., Zuo Y., Zhang P., Yang P. (2015). Sevoflurane enhances neuromuscular blockade by increasing the sensitivity of skeletal muscle to neuromuscular blockers. *Int J Physiol Pathophysiol Pharmacol*.

[17] Hayamizu K., Chaki T., Tachibana S., Hirata N., Yamakage M. (2021). Effect of remimazolam on intraoperative neuromonitoring during thyroid surgery: a case series. *Journal of Anesthesia*.

[18] Kondo T., Toyota Y., Narasaki S. (2020). Intraoperative responses of motor evoked potentials to the novel intravenous anesthetic remimazolam during spine surgery: a report of two cases. *JA Clin Rep*.

[19] Wulf H., Ledowski T., Linstedt U., Proppe D., Sitzlack D. (1998). Neuromuscular blocking effects of rocuronium during desflurane, isoflurane, and sevoflurane anaesthesia. *Canadian Journal of Anaesthesia*.

[20] Veselis R. A., Reinsel R. A., Feshchenko V. A., Wroński M. (1997). The comparative amnestic effects of midazolam, propofol, thiopental, and fentanyl at equisedative concentrations. *Anesthesiology*.

[21] Ghouri A. F., Ramirez Ruiz M., White P. F. (1994). Effect of flumazenil on recovery after midazolam and propofol sedation. *Anesthesiology*.

